# Nasopharyngeal Inflammatory Polyp With Pseudo‐Lymphangiomatous Features: A Case Report

**DOI:** 10.1155/crot/6236430

**Published:** 2026-11-04

**Authors:** Christian Woodbridge, Alejandro Fandino, Sahr Kortequee

**Affiliations:** ^1^ Department of Otolaryngology-Head and Neck Surgery Rotorua Hospital Health New Zealand | Te Whatu Ora Bay of Plenty New Zealand

**Keywords:** case report, immunohistochemistry, lymphangioma, nasal polyp, nasopharyngeal mass, pseudolymphangiomatous change

## Abstract

Nasopharyngeal masses encompass a broad differential diagnosis, including inflammatory, congenital and neoplastic lesions. Lymphangiomas are rare benign lymphatic malformations and are infrequently reported in the nasopharynx. We present an unusual case of an inflammatory nasopharyngeal polyp with pseudolymphangiomatous histological features that initially mimicked lymphangioma. A 22‐year‐old male presented with recurrent nasal obstruction. Endoscopy demonstrated an atypical polypoid lesion protruding through the left choana. Computed tomography showed a 30 × 22 × 20 mm lesion arising from the left superior turbinate and extending into the nasopharynx, without bone destruction. Endoscopic resection was performed. Initial histology showed multiple dilated fluid‐filled spaces within densely hyalinised stroma, with CD31 positivity, raising concern for lymphangioma. The ERG‐negative profile was atypical, and repeat immunohistochemistry with CD31, D2‐40 and CD34 failed to demonstrate endothelial lining of the spaces. The fluid‐filled spaces were, therefore, interpreted as stromal oedema rather than true lymphatic channels, and the diagnosis was revised to an oedematous inflammatory polyp with pseudolymphangiomatous appearance. Postoperative follow‐up was arranged, but the patient did not attend. Our targeted literature review did not identify previous reports of pseudolymphangiomatous change in inflammatory nasal polyps. This case expands the recognised histopathological spectrum of inflammatory sinonasal polyps and underscores the need for cautious clinicopathological interpretation of rare nasopharyngeal presentations.

## 1. Introduction

Inflammatory sinonasal polyps are benign, nonneoplastic lesions commonly associated with chronic rhinosinusitis with nasal polyps (CRSwNP) [1]. Although many nasal polyps are clinically straightforward, unilateral, atypical or nasopharyngeal lesions require careful assessment because the differential diagnosis includes benign congenital lesions, vascular tumours, lymphatic malformations and malignancy [2, 3].

Lymphangiomas, now more commonly referred to as lymphatic malformations, are uncommon benign lymphatic lesions. They are usually congenital and are characterised histologically by endothelial‐lined lymphatic spaces [4, 5]. Nasopharyngeal lymphangiomas are rare, particularly in adults, and have been described only in isolated case reports [6–8].

We report an inflammatory nasopharyngeal polyp with pseudolymphangiomatous morphology that initially mimicked lymphangioma but lacked endothelial lining on repeat immunohistochemistry. This case illustrates a diagnostic pitfall at the interface of otolaryngology and pathology.

## 2. Case Presentation

A 22‐year‐old male first presented to Otolaryngology in 2018 with longstanding right‐sided nasal obstruction, not associated with rhinorrhoea or nasal allergy symptoms. Examination demonstrated marked right‐sided nasal septal deviation, inflamed nasal mucosa and sphenoid and superior meatal polyps. He was treated with a one‐week course of oral prednisolone and fluticasone nasal spray twice daily for 3 months. He did not attend the planned three‐month follow‐up appointment. He returned 3 years later with improved symptoms and was discharged with an open appointment.

In 2024, he represented with recurrent nasal obstruction. Nasal endoscopy demonstrated an atypical polypoid lesion protruding through the left choana. Systemic and intranasal steroid therapy was restarted, and semiurgent computed tomography was arranged. This showed a 30 × 22 × 20 mm polypoid lesion arising from the left superior turbinate and extending into the nasopharynx, causing nasopharyngeal airway narrowing without bone destruction (Figure 1). The clinical and radiological differential diagnosis included an inflammatory or choanal polyp, retention cyst, Thornwaldt cyst, Rathke pouch remnant, benign lymphatic or vascular lesion, inverted papilloma, lymphoma and nasopharyngeal carcinoma.

**FIGURE 1:**
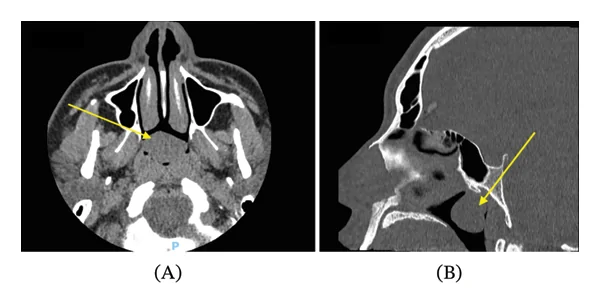
Noncontrast computed tomography of the paranasal sinuses and nasopharynx. (A) Axial soft‐tissue window demonstrating a homogeneous polypoid lesion arising from the left posterior nasal cavity/superior turbinate region and extending into the nasopharynx (arrow). (B) Sagittal bone‐window image demonstrating nasopharyngeal airway narrowing caused by the lesion (arrow), without adjacent bone destruction.

The patient proceeded to endoscopic surgery. Intraoperatively, a midline cystic/polypoid nasopharyngeal mass was identified, and the bulk of the lesion was resected. Thorwaldt cyst and a Rathke pouch remnant were considered intraoperatively because of the cystic appearance and location.

Initial histopathology reported lymphangioma, with a central stalk occupied by numerous dilated lymphatic‐type spaces containing fluid and associated submucosal lymphoid infiltrate. Immunoperoxidase staining was initially reported as CD31‐positive and ERG‐negative. Because the morphology and immunoprofile were unusual for lymphangioma, the tissue was reexamined. Repeating CD31 staining, with additional D2‐40 and CD34 immunohistochemistry, was negative in the central pseudovascular spaces, indicating the absence of an endothelial lining (Figure 2). The centre of the polyp contained densely hyalinised stroma, and the fluid‐filled spaces were interpreted as oedematous change rather than lymphatic channels. The final diagnosis was an oedematous inflammatory polyp with pseudolymphangiomatous appearance.

**FIGURE 2:**
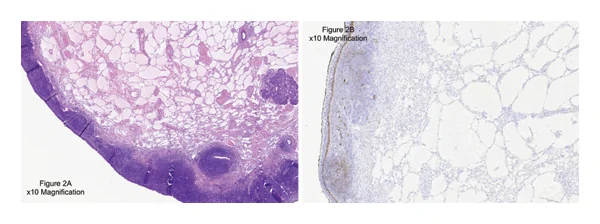
Histopathology and immunohistochemistry of the resected polyp. (A) Haematoxylin and eosin stain, × 10 magnification, showing respiratory epithelium, subepithelial lymphoid tissue and multiple dilated pseudolymphangiomatous spaces containing eosinophilic oedema fluid within hyalinised stroma. (B) D2‐40 immunohistochemistry, × 10 magnification, showing positive staining in normal lymphatic channels and absence of endothelial staining in the pseudolymphangiomatous spaces.

Postoperative nasoendoscopic follow‐up was arranged, but the patient did not attend the appointment. Consequently, formal postoperative endoscopic assessment, recurrence status and patient perspective are not available. The timeline of clinical events is summarised in Table 1.

**TABLE 1:** Timeline of clinical events.

Time	Clinical event	Management/outcome
2018	Initial presentation with longstanding right‐sided nasal obstruction. Examination showed right septal deviation, inflamed nasal mucosa, and sphenoid and superior meatal polyps.	One‐week course of oral prednisolone and fluticasone nasal spray twice daily for 3 months. Planned follow‐up was not attended.
2021	Returned with improved symptoms.	Discharged from Otolaryngology with an open appointment.
2024	Re‐presented with recurrent nasal obstruction. Endoscopy showed an atypical polypoid lesion protruding through the left choana.	Systemic and intranasal steroid therapy restarted. Semiurgent computed tomography requested.
2024	Computed tomography demonstrated a polypoid lesion arising from the left superior turbinate and extending into the nasopharynx.	Endoscopic resection arranged.
2024	Endoscopic surgery identified a midline cystic/polypoid nasopharyngeal mass.	Bulk resection performed. Histopathology initially suggested lymphangioma, then was revised after repeat immunohistochemistry.
Postoperative	Postoperative nasoendoscopic follow‐up was arranged.	Patient did not attend; formal endoscopic outcome assessment and patient perspective are unavailable.

## 3. Discussion

This case demonstrates how marked stromal oedema in an inflammatory polyp can closely mimic a lymphatic lesion. The initial diagnosis of lymphangioma was understandable because of the multiple dilated fluid‐filled spaces within the central stalk and the reported CD31 positivity. However, true lymphangioma requires endothelial‐lined lymphatic channels. The ERG‐negative profile was atypical for a vascular or lymphatic lesion, and repeat CD31, D2‐40 and CD34 staining failed to demonstrate endothelial lining of the spaces [9, 10]. These findings supported oedema within hyalinised stroma rather than true lymphatic channels.

The case is clinically relevant because adult nasopharyngeal masses have a broad differential diagnosis. Benign possibilities include inflammatory polyps, choanal polyps, Thornwaldt cyst, Rathke pouch remnant, branchial cyst, adenoid hypertrophy, juvenile nasopharyngeal angiofibroma and benign vascular or lymphatic malformations. Important malignant diagnoses include nasopharyngeal carcinoma, lymphoma and sinonasal sarcoma [2, 3]. Imaging in this case supported a benign process because there was no bone destruction, but histology and repeat immunohistochemistry were essential for definitive diagnosis.

Published nasopharyngeal lymphangioma cases remain limited. Adult nasopharyngeal lymphangioma has been reported as an unusual cause of nasal obstruction and has generally been managed surgically [6]. A further case described lymphatic malformation of the nasopharynx during pregnancy [7], and a recent adult‐onset case reported an endoscopically managed cystic nasopharyngeal lymphangioma in a 25‐year‐old woman [8]. These reports differ from the present case because they describe true lymphatic malformations, whereas the present lesion was an inflammatory polyp with pseudolymphangiomatous change.

Our targeted literature search did not identify a previous case report of pseudolymphangiomatous morphology in an inflammatory nasal polyp. This supports the novelty of the case, although the nonsystematic nature of the search means that isolated unpublished or unindexed cases cannot be excluded. As a single case report, this observation cannot establish incidence, recurrence risk or long‐term outcome. A further limitation is the absence of postoperative endoscopic follow‐up, as the patient did not attend the arranged appointment. Nevertheless, the case expands the recognised histopathological spectrum of inflammatory sinonasal polyps and highlights the need for clinicopathological correlation when unusual stromal morphology is encountered.

## 4. Conclusion

Pseudolymphangiomatous change in an inflammatory nasopharyngeal polyp can closely mimic lymphangioma. Repeat immunohistochemistry and correlation with the clinical and radiological findings were essential in distinguishing stromal oedema from true lymphatic channels. This report contributes to the limited literature on nasopharyngeal lymphatic lesions and underscores the importance of cautious pathological interpretation in rare presentations.

## Author Contributions

Christian Woodbridge contributed to data collection and drafting of the manuscript. Alejandro Fandino contributed to data interpretation and manuscript revision. Sahr Kortequee contributed to patient care, supervised the project and critically revised the manuscript.

## Funding

The authors received no financial support for the research, authorship or publication of this article.

## Ethics Statement

Ethics approval was not required for this retrospective single case report in accordance with institutional policy.

## Consent

Written informed consent was obtained from the patient for publication of this case report.

## Conflicts of Interest

The authors declare no conflicts of interest.

## Data Availability

Data sharing is not applicable to this article as no datasets were generated or analysed. Relevant clinical information is included in the article.
